# Providing actionable evidence in Public Health – The 2018 international workshop on evidence-based public health at the Robert Koch Institute, Berlin

**DOI:** 10.25646/6499

**Published:** 2020-06-04

**Authors:** Christa Scheidt-Nave, Angela Fehr, Sebastian Haller, Giselle Sarganas, Henriette Steppuhn, Julia Truthmann, Thomas Harder

**Affiliations:** 1 Robert Koch Institute, Berlin, Department of Epidemiology and Health Monitoring; 2 Robert Koch Institute, Berlin, Centre for International Health Protection; 3 Robert Koch Institute, Berlin, Department of Infectious Disease Epidemiology; 4 Formerly Robert Koch Institute, Berlin, Department of Epidemiology and Health Monitoring

A one-day international workshop entitled ‘Evidence-based Public Health for Public Health Action’ took place at the Robert Koch Institute (RKI) in Berlin on December 14, 2018. The workshop was organised by an interdisciplinary RKI public health research team and aimed to (1) provide insight into current concepts and methodological challenges in evidence-based public health (EBPH), and (2) identify next steps in enhancing collaborations on EBPH research and practice within the RKI and with external partners at the national and international level.

The workshop consisted of two parts. The first part comprised a series of invited talks given by experts in the field of EBPH from Germany and the United Kingdom (UK). The second interactive part was devoted to group discussions. Applying the world café method, participants were asked to discuss approaches to strengthen EBPH at the RKI. There was time for group discussions at three tables in two twenty minute rounds. Inspired by the public health action cycle three key questions were assigned to the table hosts in order to stimulate and guide discussions:

Table 1 (ASSESSMENT) – Which tools/methods for systematic evidence assessment does the RKI as a national public health institute need to identify and prioritize public health topics?

Table 2 (EVALUATION) – Which methods, skills, and data does the RKI as a national public health institute need to perform evaluations of public health interventions?

Table 3 (DISSEMINATION) – How can the RKI as a national public health institute facilitate dissemination of results among public health stakeholders, and what could be important steps to enhance that (e.g. Cochrane Public Health Research network, institutional repositories)?

The workshop was open to RKI staff from all units. A total of 66 persons participated including invited speakers. The workshop program is available on the publication server of the RKI.

## Part 1: Invited talks

In their introductory presentations Mark Petticrew, Department of Social and Environmental Health Research at the London School of Hygiene and Tropical Medicine, and Eva A. Rehfuess, Institute for Medical Information Processing, Biometry, and Epidemiology at the Pettenkofer School of Public Health, Ludwig Maximilian University of Munich, outlined current concepts and methodological challenges in EBPH. In particular, both speakers highlighted the fact that public health interventions always imply changes in complex systems. With a special focus on public health interventions targeting the prevention of non-communicable diseases, Marc Petticrew emphasized the need to study not only what is easy to measure, e.g. individual health behaviour, but also the upstream causes. These include personal, societal and economic context factors, such as health perceptions or market forces. Understanding the complex determinants of non-communicable diseases is essential to provide evidence for the implementation and evaluation of effective public health interventions across very different contexts. Eva A. Rehfuess illustrated the importance of contextual factors by providing examples of interventions that proved to be beneficial in one context, but harmful in another. Thus, deriving evidence from randomized controlled trials (RCTs) may result in seriously misleading results if contextual factors are not taken into account. Logic models can be used as a graphical tool for mapping contextual factors relevant to the design and evaluation of public health interventions. Several conceptual frameworks are available to guide the process from evidence synthesis to decision-making in public health.

Kay Nolan, Centre for Guidelines at the National Institute for Health and Care Excellence (NICE) in Manchester/UK, shared her expertise in EBPH guideline development. She illustrated two of the NICE core principles. First, given the complexity of public health problems, it is inevitable to systematically search for the best available evidence. This requires considering information across the whole spectrum of evidence levels. Secondly, identifying evidence gaps and areas of uncertainty is a central part of NICE EBPH guideline recommendations. This will help to guide research priority setting, in order to continuously improve the evidence base. These principles have the potential to guide the next steps to strengthen EBPH at the RKI.

Till Bärnighausen, Heidelberg Institute of Global Health (HIGH), University of Heidelberg, introduced innovative methods in population-based implementation and evaluation research including regression-discontinuity and fixed-effect models. He demonstrated that quasi-experimental study designs play a key role in public health intervention research, in particular when conduct of RCTs is precluded for ethical and methodological reasons. If carefully designed and appropriately applied to a specific research question, quasi-experimental methods minimize risk of bias and hence provide high level evidence regarding the effectiveness of public health measures.

Stefan Lhachimi, Institute for Public Health and Nursing Research (IPP), University of Bremen, discussed some of the challenges specific to the conduct of systematic reviews and meta-analyses on public health interventions. He emphasized the need for improving the quality of the evidence basis at the level of primary studies, in particular with regard to reducing risk of bias, standardizing definitions for outcomes and interventions, and considering that study results may greatly vary according to the specific study contexts. He also discussed ongoing work to improve methods in synthesizing the evidence on public health intervention, in order to ensure timely and actionable information for health policy planning and implementation.

Manfred Wildner, Department of Health in the Bavarian Health and Food Safety Authority, Oberschleißheim, and Pettenkofer School of Public Health, Ludwig Maximilian University of Munich, highlighted the need for a sustainable translation network in EBPH. Based on his longstanding experience at the interface of public health policy, research and practice, he emphasized that an ongoing, structured and open discourse involving policy, research and practice should be guided by the shared responsibility to provide the best available evidence to health policy making, to support implementation and evaluation research, and to inform the public.

Thomas Harder, Department of Infectious Disease Epidemiology, Robert Koch Institute, Berlin, illustrated two examples of EBPH research and practice at the RKI. The RKI took the lead in the PRECEPT project (Project on a Framework for Rating Evidence in Public Health). In this project, an international and multidisciplinary research team developed and successfully implemented a conceptual framework for rating evidence in public health with focus on the prevention and control of communicable diseases. The project was funded by the European Center for Disease Prevention and Control (ECDC). The German Standing Committee on Vaccination (STIKO) provides and continuously updates recommendations on vaccinations in accordance with the German Protection against Infection Act (IfSG). These recommendations are based on systematic reviews of the biomedical literature which are conducted by the STIKO Executive Secretariat at RKI. Recommendations serve to inform the public, to advise federal health authorities on vaccination policies and programs, and to support decisions on reimbursement of vaccinations within the statuary health insurance system by the Federal Joint Committee.

## Part 2: Group discussions

Group discussions at the three tables delineated several key issues with regard to enhancing next steps for strengthening EBPH at the RKI.

Table 1 (ASESSMENT) – Which tools/methods for systematic evidence assessment does the RKI as a national public health institute need to identify and prioritize public health topics?

In addition to rating evidence in public health, future work should focus on health gap analyses and priority setting, in order to generate the evidence that is presently most needed and actionable. This will also help to make efficient use of time and personnel resources. Systematic approaches to health gap analyses including quantitative as well as qualitative methods, such as discourse analysis may be necessary to achieve this goal.

Table 2 (EVALUATION) – Which methods, skills, and data does the RKI as a national public health institute need to perform evaluations of public health interventions?

There is a need to strengthen and continuously develop EBPH research methods to generate evidence in public health. This includes the use of innovative methods to abbreviate systematic literature reviews and evidence synthesis (e.g. overviews of reviews) as well as the application of methods for health impact assessment, in order to aid health policy planning and decision-making. In addition, quasi-experimental study designs could be included in the methodological repertoire to evaluate public health interventions at the population level. This would help to strengthen implementation research at the interface between public health research and practice.

Table 3 (DISSEMINATION) – How can the RKI as a national public health institute facilitate dissemination of results among public health stakeholders, and what could be important steps to enhance that (e.g. Cochrane Public Health Research network, institutional repositories)?

Timely and effective dissemination of evidence in public health is essential for the implementation of public health interventions. It requires building strong networks between research, practice and policy. It also requires harnessing methods and technologies to collate, visualize and communicate the results of evidence-based public health research to the specific user groups in need of information for action.

Overall, the workshop highlighted that principles and methods of EBPH are fundamental to advance public health research that informs and influences policy and practice, which has been defined as one of the essential public health functions by the World Health Organization. This will be necessary given new public health threats from infectious diseases and antimicrobial/antibiotic resistance as well as from an increasing burden of non-communicable diseases and age-related health conditions at the national as well as global level.

